# The Synergistic Effect of Sodium Hypochlorite (NaClO) and Organosilicone Adjuvant Enhances the Inhibition and Oxidative Damage in *Cladophora* sp.

**DOI:** 10.3390/biology14121773

**Published:** 2025-12-11

**Authors:** Liangjie Zhao, Chenxi Tan, Yongtao Tang, Zhen Zhang, Liangxin Guo, Gaoyou Yao, Qihu Dai, Yongxu Cheng, Chen Qian

**Affiliations:** 1 School of Fisheries, Xinyang Agriculture and Forestry University, Xinyang 464000, China; a850924t@163.com (L.Z.); tanchenxi2013@163.com (C.T.); t13721071655@126.com (Y.T.); zzhang@xyafu.edu.cn (Z.Z.); gaoyouyao@126.com (G.Y.); fairydqh@126.com (Q.D.); 2 Xinyang Fisheries Station, Xinyang 464000, China; xysc6653765@163.com; 3 School of Marine Sciences, Ningbo University, Ningbo 315832, China; chengyongxucrablab@hotmail.com; 4 College of Fisheries and Life Science, Shanghai Ocean University, Shanghai 201306, China

**Keywords:** sodium hypochlorite, organosilicone adjuvant, *Cladophora*, inhibitory effects, oxidative damage

## Abstract

*Cladophora*-dominated filamentous algal blooms constitute a growing threat to aquatic ecosystem stability and aquaculture operations. This study systematically evaluated the algicidal efficacy of sodium hypochlorite (NaClO), both individually and in combination with an organosilicone adjuvant, against *Cladophora* sp., with particular focus on induced oxidative damage mechanisms. Results demonstrated that NaClO exhibited a dose- and time-dependent inhibitory effect, achieving ≥90% inhibition at 1.20 mmol L^−1^ within 48 h and >99% by 120 h, significantly surpassing the efficacy of lower concentrations (0.40 mmol L^−1^) (*p* < 0.05). Physiological assessment revealed that 1.20 mmol L^−1^ NaClO significantly suppressed total antioxidant capacity (T-AOC) after 96 h (*p* < 0.05). Notably, synergistic enhancement was observed with adjuvant co-application: at 0.40 mmol L^−1^ NaClO, superoxide dismutase (SOD) activity reached its minimum with 0.33 ppm organosilicone adjuvant, showing significant reduction compared to higher adjuvant concentrations (*p* < 0.05). Furthermore, combinations of 0.80 and 1.20 mmol L^−1^ with organosilicone adjuvant consistently maintained inhibition rates ≥ 94% across all sampling intervals, achieving control efficacy equivalent to 2.00 and 2.40 mmol L^−1^ NaClO alone within 48 h. These findings demonstrate that NaClO supplemented with low-dose organosilicone adjuvant constitutes a promising and efficient strategy for mitigating *Cladophora* blooms.

## Simple Summary

Algal blooms composed of filamentous green algae, particularly *Cladophora*, pose an escalating threat to the sustainability of aquatic ecosystems. *Cladophora*, characterized by its strong competitive traits and robust defense capacity, remains a pivotal challenge in water management. To address this issue, this study explored the inhibitory effect of sodium hypochlorite (NaClO) and its synergistic effects with an organosilicone adjuvant on *Cladophora* sp. NaClO alone exhibited inhibitory effects and oxidative damage on *Cladophora* sp. in a dose- and time-dependent manner. Notably, the synergistic use of a low-dose organosilicone adjuvant significantly enhanced the inhibitory effect of NaClO. This study offers a feasible reference strategy for the control of *Cladophora* blooms and for the first time confirms that organosilicone adjuvants can enhance the inhibitory efficiency of NaClO against *Cladophora* sp., providing guidance for the development of efficient and sustainable control technologies for filamentous green algae blooms.

## 1. Introduction

Filamentous algal blooms pose a significant and escalating threat to aquatic ecosystems globally [1,2,3,4], characterized by rapid biomass accumulation [5] and the development of floating algal mats that can attain thicknesses of several tens of centimeters [6]. These proliferative mats not only physically dominate the water column [7,8], but also induce shifts in physicochemical water conditions [9], and exacerbate competition for essential nutrients [10]. Evidence demonstrates that such mats cause statistically significant alterations in the vertical profile (0–2 m), with severe dissolved oxygen (DO) depletion recorded at depths as shallow as 0.5 m [11]. Furthermore, the senescence and decomposition of this algal biomass precipitate a critical deterioration of water quality, manifesting as marked reduction in DO and pH [12,13], the liberation of toxic gases [14], the release of allelopathic compounds [15], and perturbations to biogeochemical cycling [16]. These cascading effects collectively degrade aquatic habitats, resulting in the mortality or displacement of fish and macroinvertebrate populations, and ultimately compromising entire ecological balance [17,18].

Filamentous algal mats are typically monospecific, most commonly formed by *Cladophora* [19]. This genus exhibits a suite of competitive traits, including lower temperature optima [20], higher light requirements [21], efficient nutrient uptake [22], and rapid reproductive rates [23], which confer an absolute advantage in interspecific competition. Consequently, in early spring, shallow aquaculture ponds, particularly those for shrimp and crab cultivation, provide an ideal environment for *Cladophora* proliferation, often leading to complete surface coverage by algal mats. The resultant economic losses have prompted the indiscriminate use of unregulated herbicides, which in turn cause significant aquaculture losses annually [24]. Compounding the challenge, *Cladophora* displays characteristics of a pioneer species and a notable resistance to environmental stressors [25]. Currently, few effective control strategies exist for *Cladophora* in natural water bodies. Conventional methods, including the application of heavy metal ions and herbicides, are plagued by pronounced shortcomings such as low efficiency, high maintenance demands, and substantial ecological risks [26]. While biological control measures at early growth stages have been reported [27], their efficacy remains limited during the outbreak period. Therefore, the development of a safe and effective method to control *Cladophora* blooms persists as a critical and unresolved challenge in aquatic management.

Owing to its potent oxidizing properties, sodium hypochlorite (NaClO) is widely used as a algicidal biocide for algal control [28,29]. Its efficiency is documented across various algal phyla. For instance, within the Chlorophyta, a concentration of 0.10 mg L^−1^ NaClO inhibited the growth of *Closterium ehrenbergii* within 6 h and significantly reduced its chlorophyll autofluorescence within 12 h [30]. Similarly, in tertiary treated effluent, 3.00 mg L^−1^ NaClO induced rapid cell lysis in the cyanobacterium *Microcystis aeruginosa* within 30 min [31]. Against the diatom *Phaeodactylum tricornutum* (Bacillariophyta), exposure to 0.20 mg L^−1^ NaClO in cooling wastewater rapidly suppressed photosynthesis and growth within 24 h [32]. However, the inhibition of NaClO on the robust genus *Cladophora* remains unexplored. The defensive capacity of *Cladophora* is significant, as its cell walls contain substantial cellulose and algal-specific gums that confer structural integrity [33]. More critically, the cells can deposit siliceous coatings on hard substrates, forming a mucilaginous layer with strong adsorptive affinity and exceptional resistance to disinfectants [34].

While NaClO is effective, its environmental safety, particularly in natural ecosystems beyond controlled wastewater settings, requires careful consideration. Although its use in water treatment is well-established, and ecological risks can be managed through controlled application and residual oxidant removal [35]. Nonetheless, to minimize potential ecosystem impacts, reducing NaClO dosage is highly desirable. When used at appropriate concentrations, its persistence is relatively short and the risk of long-term accumulation is low [36]. Previous studies suggest that synergistic combinations can enhance the efficacy of NaClO. For example, co-application with potassium iodide reduced lethal concentrations for protozoa compared to either agent alone [37], and elevated CO_2_ levels were shown to potentiate NaClO toxicity in certain marine phytoplankton [38]. Given the well-protected nature of *Cladophora*, we therefore sought to investigate whether a suitable synergist, specifically an organosilicone adjuvant, could potentiate NaClO to overcome its defensive mechanisms and achieve effective inhibition. Organosilicone adjuvants, such as polyether-modified trisiloxanes, are characterized by their exceptional surface activity, environmental compatibility, chemical stability, and cost-effectiveness [39]. They have been demonstrated as effective synergists in various applications [40,41], potentially enabling reduced NaClO usage while maintaining or even enhancing algicidal performance. This approach supports more environmentally sustainable management. In summary, the objectives of this study were:

1. To evaluate the inhibitory efficacy of NaClO on *Cladophora* sp.;
2. To identify a potential synergistic enhancer for NaClO-mediated inhibition of *Cladophora*;
3. To elucidate the physiological damage mechanisms in *Cladophora* induced by NaClO, both alone and in combination with a synergistic enhancer.

This research provides a foundation for optimizing existing algal control strategies and advancing the development of efficient, eco-friendly treatment technologies. It also contributes scientific evidence and practical approaches for mitigating eutrophication and managing algal blooms.

## 2. Materials and Methods

### 2.1. Experimental Material Identification

Vigorously growing filaments of *Cladophora* sp. were collected from a Chinese mitten crab (*Eriocheir sinensis*) culture pond in Chongming District, Shanghai, China (31.5784082° N, 121.5532700° E). To accurately identify the filamentous green algae samples collected from the field and to distinguish them from morphologically similar taxa within the genera *Cladophora* and *Spirogyra*, 18S rRNA gene sequencing was performed on representative samples, and a phylogenetic tree was constructed using existing green algal gene sequences. In the phylogenetic analysis, species of *Spirogyra* were included as an outgroup reference. This was primarily to ensure a clear molecular distinction between *Cladophora* and *Spirogyra*, thereby confirming that all subsequent experiments and analyses were performed exclusively on the target *Cladophora* species. The sequence has been deposited in GenBank under accession number OP345221 [42]. The phylogenetic tree has been provided in the Supplementary Material (Figure S1). Samples formed bright-green, uniseriate filaments that were occasionally branched (Figure S2). Although the phylogenetic analysis indicated that our samples belong to the genus *Cladophora* and showed high sequence similarity to *Cladophora glomerata*, they did not form a highly supported monophyletic clade with any confirmed reference sequences of *C. glomerata*. Therefore, throughout the manuscript, the studied algal material is referred to as *Cladophora* sp. to accurately reflect its identification at the genus level.

### 2.2. Experimental Design and Samples Collection

In the laboratory, the filaments were carefully cleaned of visible debris under a dissecting microscope, treated with 0.20% (*w*/*v*) potassium iodide for 1 min to remove epiphytes, and rinsed four times with ultrapure water. The purified filaments were acclimated for 48 h in aerated ultrapure water under controlled light conditions with daily water renewal before use in experiments. The ultrapure water immersion was strictly employed for surface cleaning and epibiont reduction, with no observable detrimental effects on the *Cladophora* sp. morphology or integrity [24]. Based on its wide applicability in algal cultivation and the results of our pre-experimental verification [43,44], BG11 medium, with its balanced nitrogen-to-phosphorus ratio and comprehensive trace element composition, was selected as the standard medium in this study to ensure that the algae were in a consistent and stable physiological state at the outset of the experiments. All experiments were conducted in transparent 4 L polycarbonate tanks that were cleaned and filled with 3 L of BG11 medium. The concentrations of NaClO, with a purity of 98% (Yunnan Zhiyan Biotechnology Co., Ltd., Kunming, China) in the tanks were set at 0.00, 0.40, 0.80, 1.20, 1.60, 2.00, and 2.40 mmol L^−1^, with three replicates for each concentration. Filament biomass (1.50 g), blot-dried with absorbent paper, was transferred into each tank. These tanks were then incubated in a condition-controlled incubator (Shanghai Yiheng Technology Instrument Co., Ltd., Shanghai, China) at 26 °C and 54 µmol photons m^−2^ s^−1^, with a 12 h light/12 h dark cycle. The tanks were gently shaken three times a day and randomly rearranged to minimize any variations in irradiance.

Based on the effective inhibitory concentration (A3) determined from the initial experiment, a full factorial design was implemented. Factor A comprised three levels: the effective concentration (A3) and the two immediately lower concentrations (A1 and A2). Factor B consisted of three concentrations of an organosilicone adjuvant (Shandong Lvlong Bio-technology Co., Ltd., Shandong, China): B1 = 0.03 ppm, B2 = 0.33 ppm, and B3 = 3.33 ppm. A blank control (A0) was also included, yielding ten treatment combinations denoted as A0, A1B1, A1B2, A1B3, A2B1, A2B2, A2B3, A3B1, A3B2, and A3B3. The organosilicone adjuvant used in this study was a polyether-modified trisiloxane surfactant (99%). This adjuvant exhibited a non-catalytic nature and inherent chemical stability, therefore, did not cause the decomposition of NaClO, particularly at the low concentrations used in this study [45,46,47].

Samples for biochemical analyses were collected at 48 and 96 h post-exposure for the determination of malondialdehyde (MDA), superoxide dismutase (SOD), total antioxidant capacity (T-AOC) and total protein (TP) levels. An additional sample for double-stranded DNA (dsDNA) extraction was collected at 48 h. Chlorophyll a (Chl-a) content was measured in samples harvested at 0, 24, 48, 72, 96, and 120 h. Photomicrographs were captured at 48 h using an Olympus CX33 light microscope equipped with an ILAB AL600 digital camera (Olympus, Tokyo, Japan).

### 2.3. Experimental Indexes Determination

The MDA and TP content, SOD activity, and T-AOC were determined spectrophotometrically with commercial kits (Nanjing Jiancheng Bioengineering Institute, Nanjing, China) following the manufacturer’s instructions. For total DNA extraction, fresh *Cladophora* sp. filaments were snap-frozen in liquid nitrogen and ground to a fine powder with a pre-cooled mortar and pestle. Total dsDNA was isolated using the 3S Column Environmental Sample DNA Recovery Kit (Cat. K718; Shanghai Bocai Biotechnology Co., Ltd., Shanghai, China) according to the manufacturer’s protocol. Purity and content of dsDNA were assessed with a spectrophotometer (Beijing Purkinje General Instrument Co., Ltd., Beijing, China) [48]. Chl-a was extracted and quantified as described by Khuantrairong and Traichaiyaporn [49]. Briefly, 0.10 g of freeze-dried algal powder was homogenized in 5 mL ice-cold 90% acetone and stored in the dark at −20 °C for 18 h. After filtration (0.22 µm membrane), absorbance of the supernatant was measured at 645 and 662 nm (as *A*_645_ and *A*_662_). Chl-a content was calculated with the equation:

Chl-a (µg g^−1^ wet weight) = 11.75 × *A*_662_ − 2.35 × *A*_645_.

The inhibitory rate (IR), defined as the mortality of *Cladophora* sp., was calculated from Chl-a content using the following equation:

IR (%) = (*N*_0_ − *N_s_*)/*N*_0_ × 100,

where *N*_0_ and *N_s_* are the Chl-a contents of the control group and treatment groups, respectively.

### 2.4. Statistical Analysis

Statistical analyses were performed with SPSS 19.0 software. Homogeneity of variances was verified by Levene’s test. When necessary, logarithmic transformation, square root, or arcsine transformation was applied to the above data to determine the assumptions of homogeneity of variance and normal distribution. Treatment effects were assessed by one-way ANOVA followed by least significant difference (LSD) post hoc tests. Differences were considered significant at *p* < 0.05. Data are presented as mean ± SD (*n* = 3).

## 3. Results

### 3.1. The Inhibitory Effects of NaClO on Cladophora sp.

**Figure 1:**
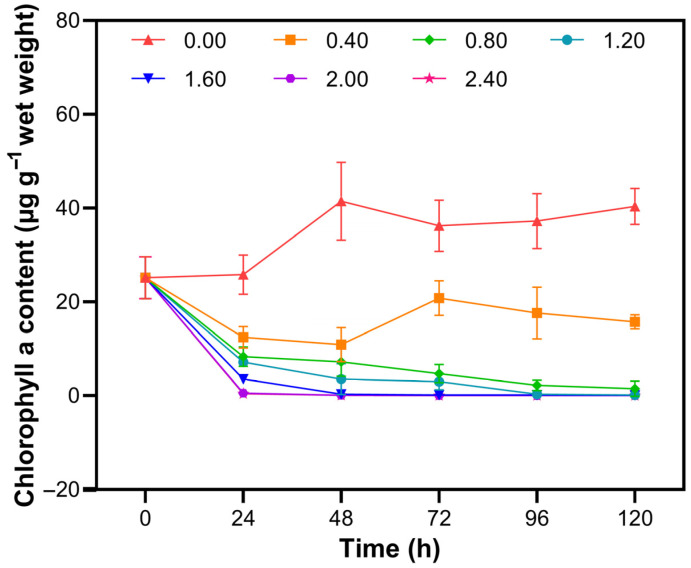
Chl-a content in *Cladophora* sp. exposed to various NaClO concentrations over time.

In the control group, the Chl-a content of *Cladophora* sp. increased steadily throughout the entire culture period (Figure 1). In contrast, exposure to 0.40 mmol L^−1^ NaClO caused a decrease in Chl-a content within the first 48 h, followed by a partial recovery. At 0.80 mmol L^−1^ NaClO, the Chl-a content decreased sharply. Under the 1.20 mmol L^−1^ treatment, the Chl-a content was virtually depleted by 96 h.

**Figure 2:**
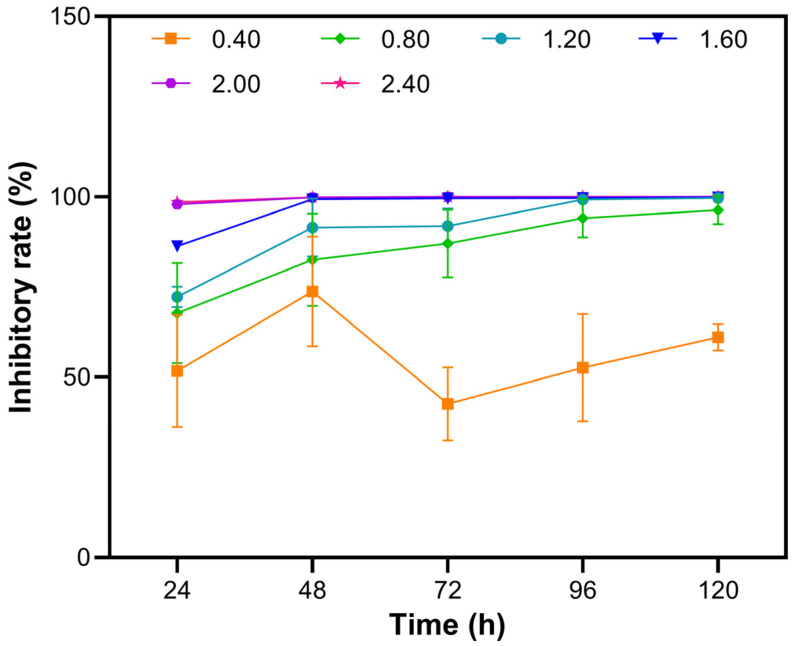
Inhibitory rates of *Cladophora* sp. exposed to various NaClO concentrations and exposure durations.

The corresponding IR curves further confirmed a distinct dose- and time-dependent inhibitory effect of NaClO on *Cladophora* sp. (Figure 2). The IR increased in an evident dose-dependent manner with increasing NaClO concentrations. For any given concentration, extending the exposure time further enhanced the inhibitory effect. Overall, significant differences were observed among the six treatment groups across all five sampling intervals (*p* < 0.05). Once the NaClO concentrations reached or exceeded 1.20 mmol L^−1^, the IR values plateaued, with no significant differences observed among these higher concentrations (*p* > 0.05). Toxicity analyses revealed 48 and 96 h LC_50_ values of 0.250 mmol L^−1^ and 0.385 mmol L^−1^, respectively (Table 1).

### 3.2. Effects of NaClO on Oxidative Damage and Antioxidant Responses in Cladophora sp.

**Figure 3:**
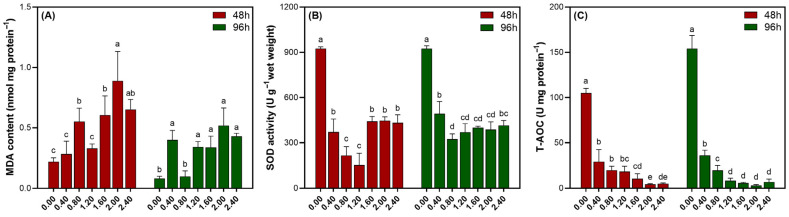
MDA content (**A**), SOD activity (**B**), and T-AOC (**C**) of *Cladophora* sp. exposed to various NaClO concentrations for 48 and 96 h. Different letters indicate significant differences (*p* < 0.05).

As depicted in Figure 3A, the MDA content in *Cladophora* sp. exhibited a unimodal response to increasing NaClO exposure at both 48 and 96 h, peaking at 2.00 mmol L^−1^ before declining. The response patterns at both time points were similar. Figure 3B illustrates the corresponding changes in SOD activity. SOD activity exhibited a biphasic response during the same time intervals, characterized by an initial decrease followed by a recovery. However, once the NaClO concentration exceeded 1.60 mmol L^−1^, the activity stabilized at a low level. It was worth noting that within the range of 0.40 to 1.20 mmol L^−1^, SOD showed a recovery at 96 h compared to 48 h. Irrespective of the exposure duration, the T-AOC of *Cladophora* sp. decreased exponentially with increasing NaClO concentrations, resulting in highly similar decay curves for the 48 and 96 h treatments (Figure 3C).

**Figure 4:**
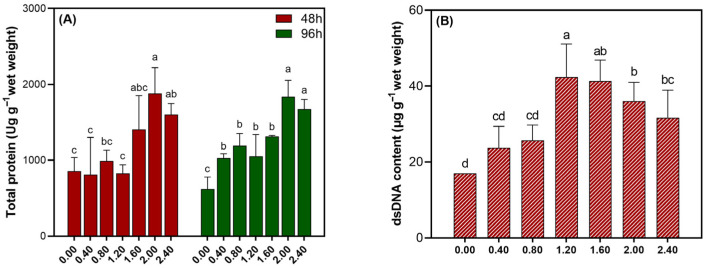
(**A**) TP content in *Cladophora* sp. exposed to various NaClO concentrations for 48 and 96 h, and (**B**) dsDNA content after 48 h of exposure. Different letters indicate significant differences (*p* < 0.05).

As illustrated in Figure 4A, the TP content of *Cladophora* sp. showed a biphasic response. Specifically, upon exposure to escalating NaClO concentrations, there was an initial increase, followed by a sharp decline, paralleling the MDA trend. A minor inflection point was observed for both parameters at 1.20 mmol L^−1^ NaClO. As shown in Figure 4B, the dsDNA content differed significantly among treatments at 48 h (*p* < 0.05). Along the NaClO concentration gradient, dsDNA content exhibited a unimodal response, attaining its peak at 1.20 mmol L^−1^. This suggested a threshold induction effect prior to substantial degradation.

### 3.3. Photomicrographs of Cladophora sp. Exposed to Various NaClO Concentrations

**Figure 5:**
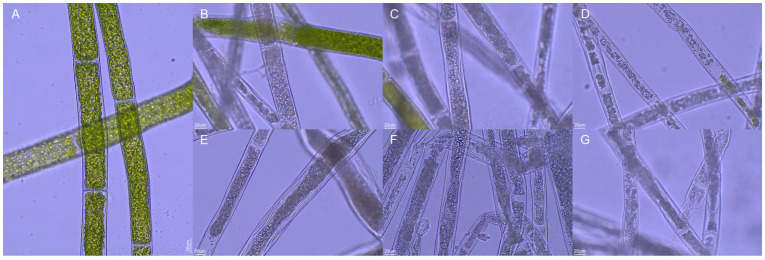
Photomicrographs of *Cladophora* sp. exposed to various NaClO concentrations for 48 h. (**A**–**G**) correspond to 0.00, 0.40, 0.80, 1.20, 1.60, 2.00, and 2.40 mmol L^−1^, respectively. The scale bar in represents 20 µm and applies to all panels.

Figure 5 depicts the micro-morphological changes of *Cladophora* sp. following 48 h of exposure to different NaClO concentrations. In the control group, the filaments maintained an intact cellular architecture, characterized by well-defined contours, evenly distributed cytoplasm, and vivid green, granular chloroplasts that were regularly arranged along the inner periclinal wall (Figure 5A). With the progressive increase in NaClO concentration (Figure 5B–G), the cells exhibited gradual cytoplasmic condensation, chloroplasts bleaching and detachment from the cell wall, culminating in cellular distortion and lysis—morphological hallmarks of oxidative injury.

### 3.4. The Inhibitory Effects of NaClO Combined with Organosilicone Adjuvant on Cladophora sp.

**Figure 6:**
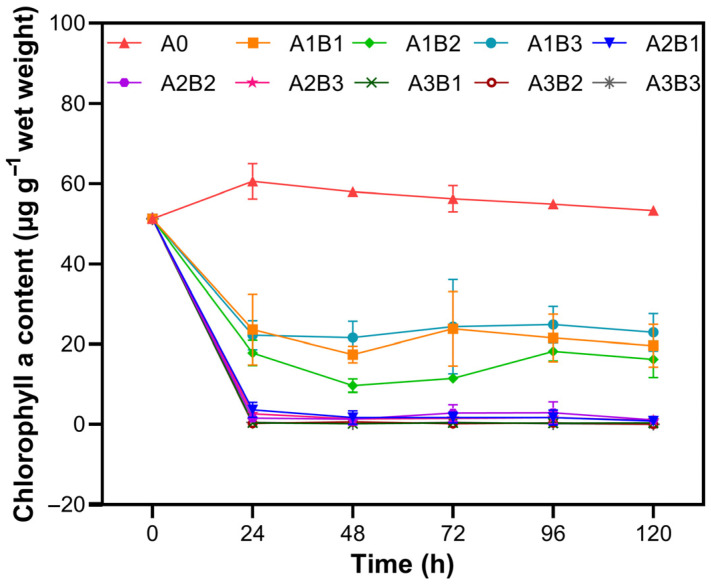
Chl-a content of *Cladophora* sp. exposed to various NaClO and organosilicone adjuvant concentrations over time.

In light of the foregoing results, a full-factorial experiment was carried out at three NaClO concentrations: A1 = 0.40 mmol L^−1^, A2 = 0.80 mmol L^−1^, and A3 = 1.20 mmol L^−1^. The Chl-a content in the control group peaked at 24 h and remained at an elevated level subsequently. In contrast, the Chl-a content in all treated groups decreased. At level A1, the Chl-a content dropped abruptly within 24 h to approximately 20 µg g^−1^ wet weight and then stabilized. The Chl-a content in groups A1B1 and A1B3 was similar and significantly higher than in A1B2. Levels A2 and A3 exhibited nearly identical responses, with the Chl-a content falling to near zero within the first 24 h (Figure 6).

As shown in Table 2, all treatment combinations within the A2 and A3 levels maintained an IR of at least 94% at every sampling point. At the A1 level, A1B2 had a higher IR compared to A1B1 and A1B3. However, these three sub-groups showed a slight decrease in efficacy over time. In contrast, both the A2 and A3 levels sustained consistently high inhibition rates (≥94%) throughout the entire incubation period.

### 3.5. Effects of NaClO Combined with Organosilicone Adjuvant on Oxidative Damage and Antioxidant Responses in Cladophora sp.

**Figure 7:**
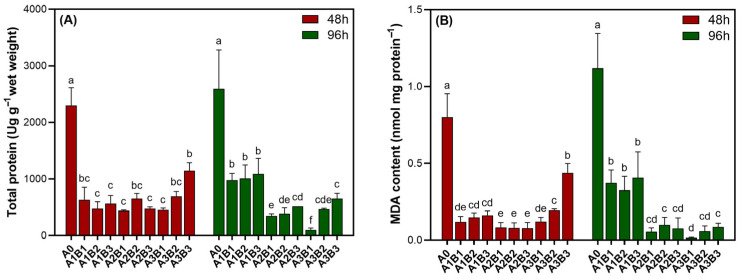
TP (**A**) and MDA (**B**) content of *Cladophora* sp. exposed to combinations of NaClO and organosilicone adjuvant for 48 and 96 h. Different letters indicate significant differences (*p* < 0.05).

The contents of TP and MDA in the A1B1 group, as illustrated in Figure 7, declined sharply and then stabilized at the A1 level, with no significant differences among the three sub-groups (*p* > 0.05). Across the NaClO gradient, both parameters decreased to their minimum values at the A2 level and then rebounded at the A3 level. The temporal profiles for 48 and 96 h were largely parallel, although the magnitude of change was notably greater at 96 h.

**Figure 8:**
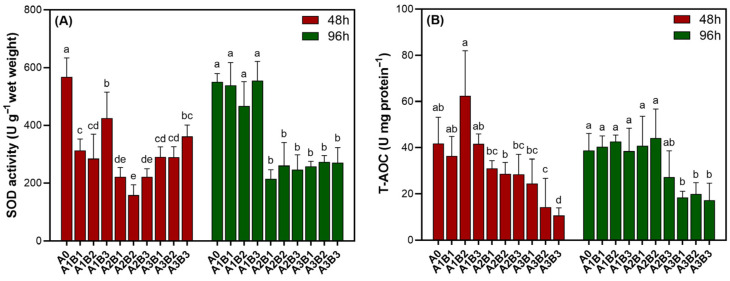
SOD activity (**A**) and T-AOC (**B**) of *Cladophora* sp. exposed to combinations of various NaClO and organosilicone adjuvant concentrations for 48 and 96 h. Different letters indicate significant differences (*p* < 0.05).

Figure 8A depicts an identical response pattern of SOD activity in *Cladophora* sp. to all treatment combinations at both 48 and 96 h. At the A1 level, SOD activity was markedly suppressed, with the most pronounced reduction observed at 48 h. Among the three subgroups, A1B2 exhibited the lowest activity, which was significantly lower than that of A1B3 (*p* < 0.05). Although higher adjuvant concentrations were tested, the lowest SOD activity was observed at 0.40 mmol L^−1^ NaClO combined with 0.33 ppm adjuvant, representing a significant reduction (*p* < 0.05). As shown in Figure 8B, T-AOC in *Cladophora* sp. decreased monotonically with increasing NaClO–organosilicone combinations at both 48 and 96 h and did not show the “V-shaped” rebound seen in SOD activity during the transition from A2 to A3. Notably, within the A1 level, the A1B2 combination displayed the lowest SOD activity while simultaneously showing the highest T-AOC value.

## 4. Discussion

Photosynthetic activity serves as a critical indicator for assessing the physiological impairment of algae [50]. Algicides can impair the photosynthetic apparatus through multiple mechanisms. These include the degradation of photosynthetic pigments, suppression of photosynthesis-related gene expression, and inhibition of key enzyme activities. Furthermore, direct damage to the integrity of thylakoid membranes and interference with the electron transport chain are also critical pathways [26,51]. Specifically, damage to Chl-a has been shown to directly compromise photosynthetic function and ultimately lead to algal cell death [52]. Consistent with these mechanisms, previous studies report that chlorine at concentrations exceeding 0.10 or 0.50 mg L^−1^ significantly depletes Chl-a and carotenoid levels [30]. The inhibitory effect of NaClO is further reflected in altered chlorophyll fluorescence parameters and modulated antioxidant enzyme activities [53]. Notably, even after residual NaClO became undetectable in the culture medium after 6 h of exposure, neither cellular density nor Chl-a content recovered after 72 h, indicating a persistent algicidal effect [29]. In the present study, NaClO exerted pronounced inhibition of *Cladophora* sp. at 0.40 mmol L^−1^, with inhibitory rates exceeding 50% at all points except 72 h. Increasing the concentration to 1.20 mmol L^−1^ resulted in consistently high inhibition (≥90%). Co-application with an organosilicone adjuvant markedly enhanced the algicidal effect: all NaClO–organosilicone combinations outperformed NaClO alone, with the A2B1 formulation sustaining ≥96% inhibition after 48 h. Interestingly, at a fixed NaClO concentration, maximal inhibition was achieved with the lowest organosilicone dosage (B2), while higher adjuvant levels (B3) reduced the suppressive efficacy. These results strongly indicate that a low-dose organosilicone adjuvant potentiates the algicidal activity of NaClO against *Cladophora* sp., likely by enhancing the penetration and bioavailability of the biocide. The reduced efficacy at higher adjuvant concentrations might be due to excessive foaming or micelle formation that could sequester NaClO, reducing its effective concentration.

MDA is an important indicator reflecting oxidative damage to cellular membrane systems [54]. Its intracellular accumulation can be induced by various stressors [55]. Elevated MDA levels consequently indicate oxidative degradation of polyunsaturated fatty acids in cellular membranes [56]. Previous investigations have documented complex dynamics in MDA accumulation under different stress conditions. For instance, Sinha et al. [57] observed in *Pistia stratiotes* L. under chromium stress that MDA content initially correlated positively with metal accumulation after 48 h, but this relationship reversed to negative after 144 h. Similarly, Tang et al. [24] reported that berberine exposure induced a characteristic biphasic response in *Cladophora* sp., where MDA content initially increased then sharply declined with rising berberine concentrations. In contrast to these temporal dynamics, NaClO exposure in our study induced a fundamentally different pattern in *Cladophora* sp. Despite its potent inhibitory effects, MDA content showed a generally monotonic increase with rising NaClO concentrations, with only a minor reduction observed at the highest concentration (2.40 mmol L^−1^). This divergence suggests distinct mechanisms of cellular damage between NaClO and previously studied inhibitors. NaClO enters the cells primarily through permeation, causing relatively mild direct damage to structural integrity. It acts mainly on intracellular components, such as chloroplasts and the mitochondrial membrane system, inducing oxidative damage and promoting MDA accumulation [58]. Conversely, other inhibitors may initiate direct membrane damage, rapidly generating substantial MDA until critical membrane failure occurs, leading to the leakage of cellular analytes into the extracellular environment. Notably, the “NaClO + organosilicone” combination treatment exhibited significantly lower intracellular MDA content despite achieving similar inhibitory efficacy to NaClO alone. This finding can be explained by the membrane-permeabilizing properties of the organosilicone adjuvant [59], which potentially facilitates enhanced diffusion of intracellular MDA to the external environment. This observation indirectly corroborates the synergistic role of organosilicone adjuvant in potentiating NaClO efficacy against *Cladophora* sp. through altered membrane permeability dynamics.

Membrane lipids and proteins, being particularly vulnerable to free radical attack, serve as reliable indicators of oxidative stress in plants [60]. In the present study, both experimental series revealed a consistent response pattern: TP and MDA contents showed strongly concordant fluctuation trends, suggesting parallel oxidative damage to both lipid and protein cellular components. This correlation may indicate the activation of a compensatory protein synthesis mechanism in *Cladophora* sp. under oxidative stress conditions. Notably, while the temporal pattern of TP content remained generally consistent between 48 h and 96 h exposures, the amplitude of variation was significantly amplified at the later time point. This difference in response magnitude over time can be explained by the concentration-dependent severity of the oxidative stress. Under sublethal NaClO concentrations, prolonged stress potentially triggered adaptive protein synthesis, whereas at higher concentrations, the cellular regulatory threshold was exceeded, resulting in progressive protein denaturation and degradation. Consequently, protein content demonstrated a biphasic regulatory response, showing accumulation under moderate stress but significant depletion under severe oxidative conditions, ultimately manifesting as enhanced response magnitude with prolonged exposure duration.

Exogenous chemical stressors can disrupt the antioxidant enzyme system in *Cladophora* cells, including excessive production of free radicals and consequent oxidative cellular damage [61]. As the primary defense against reactive oxygen species (ROS) [62], SOD demonstrates upregulated activity under mild or transient oxidative stress, with suppression occurring under more severe or prolonged exposure conditions [63]. Contrary to this established “low-promotion and high-inhibition” paradigm, our experimental data revealed that even minimal NaClO concentrations significantly suppressed SOD activity. Notably, SOD activity showed a slight recovery at moderate concentrations from 48 to 96 h. This rebound likely represents a successful adaptive physiological response, where the cell upregulates antioxidant defenses, such as synthesis of new SOD enzymes or activation of alternative repair pathways, to counteract moderate oxidative stress, highlighting the alga’s resilience and defining a sub-lethal threshold where defense mechanisms remain functional. This atypical response pattern not only indicates the potent oxidative effects of NaClO in compromising SOD function, but also reflects a capacity for rapid physiological adaptation and repair mechanisms in *Cladophora* sp. [25]. However, as NaClO concentration exceeds a critical threshold (≥1.60 mmol L^−1^), this compensatory mechanism is overwhelmed, and persistent low level of SOD activity marks the transition from reversible stress to irreversible toxicity. The sharp decline in SOD activity resulted from a dual mechanism: direct oxidative damage to existing enzymes (e.g., oxidation of the Fe^3+^ cofactor in Fe-/Mn-SOD and protein carbonylation) coupled with a failure in cellular synthesis function [64]. The disruption of cell integrity (Figure 5) suppresses the synthesis of new functional SOD enzymes, a notion supported by the declining dsDNA content (Figure 4B). This dual mechanism ultimately leads to irreversible loss of SOD activity. Moreover, the addition of the organosilicone adjuvant induced this state of irreversible damage, evidenced by unrecovered SOD activity (Figure 8A), at a significantly lower NaClO concentration (0.80 mmol L^−1^), underscoring its role in enhancing penetrability and algicidal efficiency.

To mitigate oxidative stress, plants have developed a comprehensive enzymatic and non-enzymatic antioxidant defense network, incorporating enzymes such as SOD, catalase, peroxidase, and polyphenol oxidase, along with non-enzymatic components including ascorbic acid, glutathione, and tocopherol [65,66]. T-AOC consequently serves as an indicator for assessing cellular redox homeostasis. In the present study, *Cladophora* exhibited a pronounced, concentration-dependent decline in T-AOC following NaClO exposure. Although a slight rebound in the activity of certain antioxidant enzymes was noted, this compensatory response proved insufficient to counteract the overarching suppression of total antioxidant capacity, indicating systemic impairment of the algal antioxidant system by NaClO. This response pattern contrasts markedly with the “decline followed by recovery” trajectory of T-AOC reported in *Cladophora* sp. under berberine stress [24], underscoring the more pronounced disruptive effect of NaClO on the antioxidant defense machinery. Furthermore, the consistent suppression of T-AOC observed in the full-factorial NaClO-organosilicone experiment corroborates the potent and consistent oxidative properties of NaClO in overwhelming the antioxidant barrier of *Cladophora* sp.

Lipid peroxidation of the plasma membrane drives intracellular MDA accumulation, which subsequently promotes cross-linking and polymerization of vital macromolecules including proteins and nucleic acids [67]. More critically, excessive ROS generated by exogenous chemical agents can directly induce persistent DNA lesions, constituting a primary mechanism of genotoxicity [68]. In this study, NaClO exposure elicited a biphasic response in double-stranded DNA content: concentrations ≤ 1.20 mmol L^−1^ significantly enhanced DNA synthesis, suggesting potential stress-induced replication or repair activation, whereas elevated concentrations exceeding this threshold resulted in substantial DNA degradation. This concentration-dependent transition indicates that higher NaClO levels overwhelm cellular repair mechanisms and directly compromise genomic integrity.

In the study, the organosilicone adjuvant can be hypothesized to serve as an efficient delivery system that significantly enhances the efficiency of NaClO solution penetration through its unique physical properties, including surface tension reduction, spreading capability, and stomatal penetration promotion [69,70]. This may facilitate more efficient and uniform oxidative damage to cellular structures, induction of oxidative stress, and disruption of metabolic processes, resulting in faster and more complete algal eradication of *Cladophora* sp. [29,71,72]. However, this proposed mechanism requires further validation through targeted investigations. Future research should employ more specific techniques, such as using confocal microscopy with fluorescent probes to directly visualize the penetration pathways of the adjuvant and the intracellular distribution of NaClO. Furthermore, transcriptomic and metabolomic analyses could provide comprehensive evidence for the proposed disruption of metabolic pathways at the molecular level.

Based on the current findings, the combined use of NaClO and an organosilicone adjuvant demonstrates potential for controlling established *Cladophora* mats, with surface application in algae-dense areas likely yielding better results. The organosilicone adjuvant enhances penetrability and is expected to improve control efficiency while minimizing impacts on non-target organisms. This approach could be considered for emergency treatment of filamentous algae in urban landscape waters and shows promise for application during the preliminary preparation of aquaculture ponds to mitigate bloom risks. It should be noted that the current conclusions are derived from controlled laboratory conditions. In practical applications, careful consideration must be given to the potential influence of variable environmental parameters in natural water bodies, such as pH, hardness, turbidity, organic matter content, and microbial communities, as well as the possible toxicity and long-term ecological effects on non-target organisms. Further field trials and pilot-scale studies tailored to specific aquatic environments are necessary to comprehensively evaluate the practical applicability of this technology.

Beyond efficacy and environmental safety, economic feasibility is critical for the adoption of algal control strategies in aquaculture and water management. The addition of an organosilicone adjuvant reduces the effective NaClO concentration to 0.80 mmol L^−1^ while maintaining an inhibition rate of ≥94%, reaching 97% within 48 h, thereby decreasing chemical usage and treatment frequency. The adjuvant may improve penetration and distribution uniformity, potentially allowing localized treatment of algal blooms with lower chemical doses. In contrast, as shown in Table S1, copper-based algaecides, though low in unit cost, require repeated applications due to rapid precipitation and loss of bioavailability, leading to higher long-term costs and risks of metal accumulation. Hydrogen peroxide, while environmentally benign, demands higher concentrations and more frequent dosing against filamentous algae, increasing operational expenses. Although site-specific factors such as water volume, bloom density, and environmental conditions will affect actual costs, the NaClO–organosilicone approach offers an economically promising option for controlling *Cladophora* blooms.

## 5. Conclusions

This study demonstrates that NaClO exerts its algicidal effect on *Cladophora* sp. primarily by inducing oxidative stress, leading to the functional collapse of critical physiological processes, including photosynthesis and antioxidant defenses. The co-application of an organosilicone adjuvant markedly potentiated this effect via a synergistic interaction, enabling high inhibitory efficacy at reduced NaClO dosages. The collective physiological and biochemical evidence, as indicated by the suppression of SOD and T-AOC, the accumulation of MDA, and the onset of DNA damage, confirms oxidative damage as the principal mechanism of toxicity. These findings validate the NaClO–organosilicone combination as a scientifically sound strategy with significant potential for practical application in mitigating *Cladophora* blooms. Although the NaClO–organosilicone adjuvant combination rapidly inhibits *Cladophora* sp., its routine use in aquaculture ponds requires further field validation and the establishment of species-specific safety thresholds. Future research should focus on strategies for mitigating toxicity to aquatic organisms to safeguard cultured species and microbial ecosystem functions.

## Figures and Tables

**Table 1:** Inhibitory rates of *Cladophora* sp. exposed to various NaClO concentrations over time and the corresponding LC_50_.

Groups	Inhibitory Rate of *Cladophora* sp. at Different Durations (%)				
	24 h	48 h	72 h	96 h	120 h
0.40	51.72 ± 15.60 ^d^	73.76 ± 15.25 ^c^	42.54 ± 10.16 ^c^	52.64 ± 14.90 ^b^	61.01 ± 3.74 ^b^
0.80	67.72 ± 13.91 ^c^	82.54 ± 12.81 ^bc^	87.07 ± 9.39 ^b^	94.05 ± 5.35 ^a^	96.32 ± 3.96 ^a^
1.20	72.27 ± 2.89 ^bc^	91.45 ± 7.99 ^ab^	91.85 ± 4.88 ^ab^	99.23 ± 0.73 ^a^	99.68 ± 0.25 ^a^
1.60	86.29 ± 0.56 ^ab^	99.29 ± 0.68 ^a^	99.57 ± 0.72 ^a^	99.63 ± 0.04 ^a^	99.82 ± 0.28 ^a^
2.00	97.90 ± 0.24 ^a^	99.78 ± 0.27 ^a^	99.97 ± 0.06 ^a^	99.92 ± 0.14 ^a^	99.97 ± 0.05 ^a^
2.40	98.47 ± 0.77 ^a^	99.82 ± 0.25 ^a^	99.98 ± 0.03 ^a^	100.00 ± 0.00 ^a^	100.00 ± 0.00 ^a^
LC_50_	0.454	0.250	0.445	0.385	0.350

Within the same column, different letters indicate significant differences in the inhibitory rates of *Cladophora* sp. among different concentrations at the same time point (*p* < 0.05).

**Table 2:** Inhibitory rates of *Cladophora* sp. exposed to combinations of various NaClO and organosilicone adjuvant concentrations over time.

Groups	Inhibitory Rates of *Cladophora* sp. at Different Durations (%)				
	24 h	48 h	72 h	96 h	120 h
A1B1	61.03 ± 14.64	69.92 ± 3.67	57.64 ± 16.58	60.80 ± 10.90	63.24±10.03
A1B2	70.64 ± 5.19	83.34 ± 2.93	79.62 ± 1.79	66.78 ± 4.44	69.58 ± 8.53
A1B3	63.32 ± 5.98	62.67 ± 7.08	56.68 ± 20.93	54.61 ± 8.38	56.92 ± 8.84
A2B1	94.01 ± 3.14	97.11 ± 2.92	96.94 ± 2.43	96.94 ± 3.42	98.51 ± 1.07
A2B2	97.38 ± 0.20	97.75 ± 2.52	94.94 ± 3.76	94.71 ± 5.04	97.91 ± 0.26
A2B3	95.71 ± 0.70	97.68 ± 1.36	97.38 ± 2.36	96.91 ± 2.16	98.26 ± 0.64
A3B1	99.41 ± 0.40	99.74 ± 0.24	99.37 ± 0.39	99.42 ± 0.32	99.34 ± 0.82
A3B2	99.60 ± 0.26	98.93 ± 1.50	99.69 ± 0.24	99.39 ± 0.64	99.95 ± 0.02
A3B3	99.24 ± 0.80	99.70 ± 0.35	99.15 ± 0.63	99.76 ± 0.20	99.97 ± 0.03

## Data Availability

Data are contained within the article.

## Supplementary Materials

The following supporting information can be downloaded at: https://www.mdpi.com/article/10.3390/biology14121773/s1, Figure S1. Phylogenetic tree of 18S rRNA sequences. The sequences determined in this study are shown in red. OP345221 G is the *Cladophora* sp. used in this study, and OP345222 S1 and OP348425 S2 are samples of *Spirogyra* collected from the pond. Figure S2. The photos of branching conditions of the *Cladophora* sp. used in this study. Table S1. Comparison of treatment costs and operational parameters for common *Cladophora* sp. control methods [73,74,75,76,77,78,79,80].

## Abbreviations

The following abbreviations are used in this manuscript:

NaClO	Sodium hypochlorite
T-AOC	Total antioxidant capacity
SOD	Superoxide dismutase
MDA	Malondialdehyde
TP	Total protein
Chl-a	Chlorophyll a
IR	Inhibition rate
LSD	Least significant difference
dsDNA	Double-stranded DNA
ROS	Reactive oxygen species
DO	Dissolved oxygen
