# Supplementary material for: The Synergistic Effect of Sodium Hypochlorite (NaClO) and Organosilicone Adjuvant Enhances the Inhibition and Oxidative Damage in Cladophora sp
Source: Biology (Basel). 2025 Dec 11;14(12):1773. doi: 10.3390/biology14121773 (PMC12730257; doi:10.3390/biology14121773)

Table S1. Comparison of treatment costs and operational parameters for common *Cladophora* sp. control methods [73–80].

Treatment method	Typical effective concentration	Estimated cost per unit area (USD/ha, calculated for a water depth of 1 m) *	Application frequency	Environmental and operational notes	References
NaClO alone	1.60–2.40 mmol L^{-1}	314.4–471.6	Annually or semiannually	May require neutralization, higher doses needed for robust algae.	This study
NaClO + Organosilicone adjuvant	0.80–1.20 mmol L^{-1} + 0.33 ppm	253.5–489.3	Annually or semiannually and local application as needed	Reduced NaClO dose; improved penetration; lower chemical input.	This study
Copper sulfate ($CuSO_4$)	0.1–0.3 mg L^{-1}	12.8–38.3	Multiple per season	Low initial cost but accumulates in sediments; affected by water quality (especially alkalinity); toxic to non-target organisms; frequent reapplication needed; food safety risks.	[73–75]
Hydrogen peroxide (H_2O_2)	4–15 mg L^{-1}	640.5–2403.5	Multiple per season	Rapid decomposition; low residue risk; high dosage and frequent application increase cost.	[76–80]
Mechanical/Physical removal	N/A	1697.0–3181.9+	As needed	High labor and equipment costs; immediate effect but no long-term control; often used in combination with chemicals.	/

* Cost estimates are approximate and based on typical application rates in aquaculture/pond settings; actual expenses may vary with regional pricing, water volume, and bloom severity. The current exchange rate is approximately 1 USD equals 7.0723 CNY.

Figure S1. Phylogenetic tree of 18S rRNA sequences. The sequences determined in this study are shown in red. OP345221 G is the *Cladophora* sp. used in this study, and OP345222 S1 and OP348425 S2 are samples of *Spirogyra* collected from the pond.

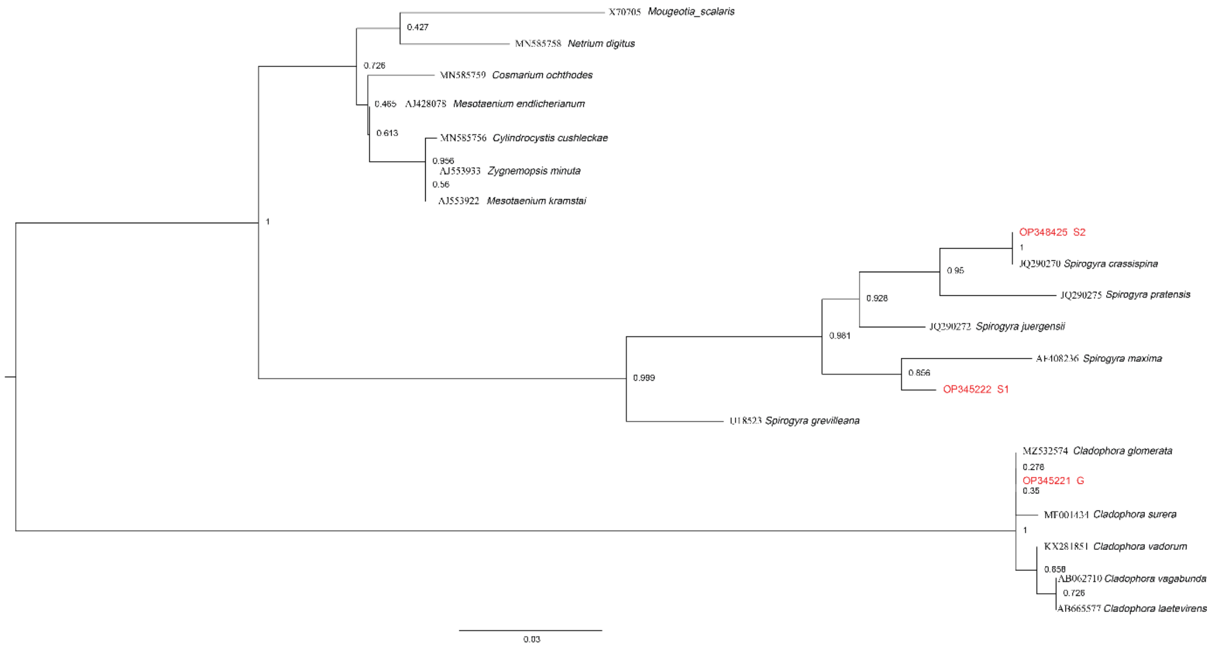

Figure S2. The photos of branching conditions of the *Cladophora* sp. used in this study.

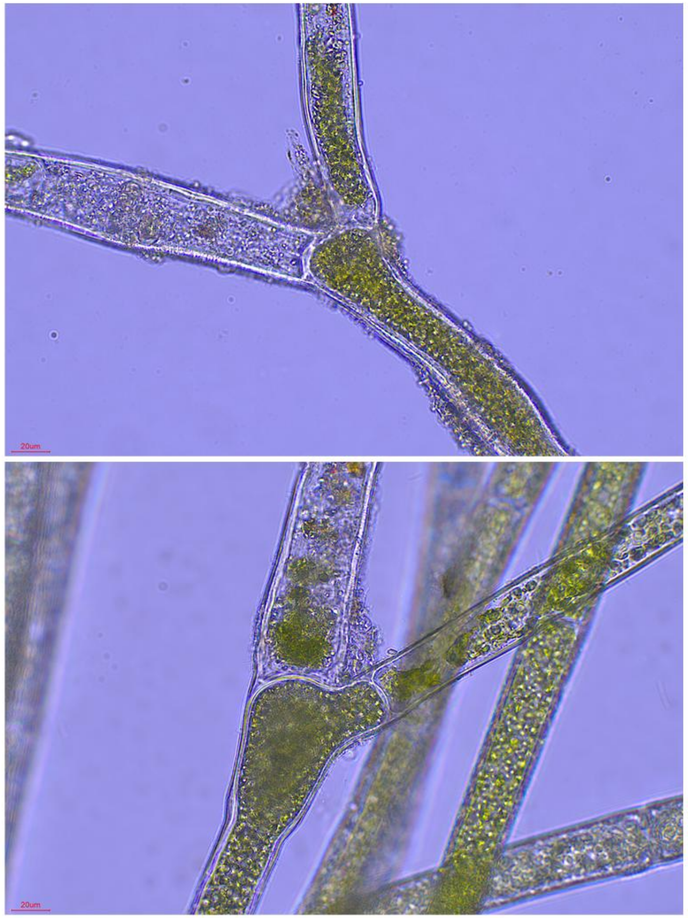

Supplement: Supplementary file 1 [file biology-14-01773-s001.zip › biology-3997020-supplementary.pdf]
